# Case of Extreme Diffusion of Odors

**Published:** 1846-06

**Authors:** 


*Case of Extreme Diffusion of Odors.*—Dr. Wilcocks, in the Philadelphia Medical Examiner, states the following fact:

When at sea, two years ago, two hundred miles to the westward of the coast of Ireland, myself and many of the other passengers observed a smoky odor, which lasted during several successive days. An appeal was made to our captain for an explanation of the phenomenon. He informed us that he had frequently remarked it before, and that it was owing to the long continuance of the easterly winds, which carried the odor of *burning peat* far out to sea.

